# Association of Body Composition with Curve Severity in Children and Adolescents with Idiopathic Scoliosis (IS)

**DOI:** 10.3390/nu8020071

**Published:** 2016-01-28

**Authors:** Edyta Matusik, Jacek Durmala, Pawel Matusik

**Affiliations:** 1 Department of Rehabilitation, School of Health Sciences, Medical University of Silesia, Ziolowa 45-47, 40-635 Katowice, Poland; ematusik@wp.pl (E.M.); jdurmala@gmail.com (J.D.); 2 Department of Pediatrics and Pediatric Endocrinology, School of Medicine in Katowice, Medical University of Silesia, Medykow 16; 40-752 Katowice, Poland

**Keywords:** idiopathic scoliosis, anthropometry, body composition, percent body fat, spinal deformity

## Abstract

The link between scoliotic deformity and body composition assessed with bioimpedance (BIA) has not been well researched. The objective of this study was to correlate the extent of scoliotic-curve severity with the anthropometrical status of patients with idiopathic scoliosis (IS) based on standard anthropometric measurements and BIA. The study encompassed 279 IS patients (224 girls/55 boys), aged 14.21 ± 2.75 years. Scoliotic curve severity assessed by Cobb’s angle was categorized as moderate (10°–39°) or severe (≥40°). Corrected height, weight, waist and hip circumferences were measured and body mass index (BMI), corrected height *z*-score, BMI *Z*-score, waist/height ratio (WHtR) and waist/hip ratio (WHR) were calculated for the entire group. Body composition parameters: fat mass (FAT), fat-free mass (FFM) and predicted muscle mass (PMM) were determined using a bioelectrical impedance analyzer. The mean Cobb angle was 19.96° ± 7.92° in the moderate group and 52.36° ± 12.54° in the severe group. The corrected body heights, body weights and BMIs were significantly higher in the severe IS group than in the moderate group (*p* < 0.05). Significantly higher FAT and lower FFM and PMM were observed in the severe IS group (*p* < 0.05). The corrected heights and weights were significantly higher in patients with severe IS and normal weight (*p* < 0.01). Normal and overweight patients with a severe IS had significantly higher adiposity levels assessed by FAT, FFM and PMM for normal and BMI, BMI *z*-score, WHtR, FAT and PMM for overweight, respectively. Overweight IS patients were significantly younger and taller than underweight and normal weight patients. The scoliotic curve severity is significantly related to the degree of adiposity in IS patients. BMI *z*-score, WHtR and BIA seem to be useful tools for determining baseline anthropometric characteristics of IS children.

## 1. Introduction

Idiopathic scoliosis (IS) is the most common form of spinal deformity in the developmental period. The main problem in this group of patients is the possibility of deformity progression. IS pathogenesis probably has a multifactorial background [1,2,3,4,5,6] and is still under debate [7]. However, recently published data showed that body composition may be an important factor in IS development [8,9]. Correct body composition, consisting of both adipose tissue and fat free mass, is essential for normal growth and stabilization of the skeletal system, especially the vertebral column. The nutritional status in children is normally assessed by means of height, weight and body mass index (BMI). The obtained results have to be interpreted according to the percentile charts for each parameter. However, this way of anthropometrical analysis does not assess the body composition and adipose tissue distribution. The results of a longitudinal study suggest that changes in BMI percentile may not accurately reflect changes in adiposity in children over time, particularly in adolescents and children with lower BMI [10]. The majority of studies performed in scoliotic children indicate that their BMI is lower than in the healthy population [11,12,13]. Today, one of the most important health problems in children and adolescents is the obesity epidemic [14,15,16,17,18,19]. The first data documenting the relationship between overweight and scoliotic curve severity in children with IS has recently been published [8,20,21]. Previous studies in IS children and adolescents were simply based on standard anthropometrical parameters (height, weight, BMI) or body composition analysis by skin fold measurement, which is a technically difficult and very subjective method [11,22,23,24,25,26]. Currently, a noninvasive body composition assessment technique is available, based on bioelectrical impedance analysis (BIA). A good correlation between BIA and dual-energy-X-ray absorptiometry (DXA) has been reported in estimating adiposity in the different groups of patients [27,28]. BIA is relatively simple, quick, non-invasive and readily accessible compared to other more sophisticated methods, such as quantitative computed tomography (qCT) or DXA. A more widespread use of DXA in children is limited mainly by its costs and low availability. The process of BIA validation resulted in the development of standards and centile charts for healthy children [29]. Our recently published preliminary report showed a significant correlation between BIA parameters and curve severity, and revealed the potential usefulness of the method in the assessment of IS population [8]. However, the limitation of our previous study was a relatively small group of patients, especially in the overweight subgroup. Therefore, further study showing the interrelationship between every anthropometrical categories of IS (underweight, normal, overweight) in relation to the severity of the deformity is needed.

Accordingly, the aim of this study was to assess the anthropometrical status of children and adolescents with IS based on standard anthropometric measurements (corrected height, weight, BMI), adjusted for age and sex corrected height *z*-score and BMI *z*-score, fat tissue distribution ratios (WHR, WHtR) and body composition parameters based on bioelectrical impedance analysis (BIA) in relation to the scoliotic curve severity.

## 2. Materials and Methods

### 2.1. Studied Population

The study group comprised 279 newly-diagnosed IS patients (224 girls/55 boys), aged 14.21 ± 2.75 years, recruited consecutively during their first visit to our Scoliosis Clinic at the Department of Rehabilitation. The diagnosis was confirmed by both, clinical assessment and standard standing postero-anterior X-ray film of the spine with Cobb’s angle ≥10°. Subjects with a history of any forms of prior treatment for scoliosis, neuromuscular diseases, endocrine diseases, skeletal dysplasia, connective tissue abnormalities, glucocorticoid therapy, fractures, mental retardation or other congenital deformities were excluded from the study.

### 2.2. Scoliotic Curve Evaluation

Scoliosis magnitude was evaluated by measuring Cobb’s angle at the coronal plane of the whole spine on a standard X-ray film. In the case of double or triple scoliotic curves, the Cobb’s angle of the major curve was selected. Curve severity was categorized as moderate (Cobb’s angle 10°–39°) or severe (Cobb’s angle ≥40°) according to the conventional classification [30].

### 2.3. Anthropometric Measurements

A set of anthropometric measurements was recorded at the first clinical visit. Standing height was measured by a wall-mounted Harpender stadiometer to the nearest 0.1 cm. Weight (in underwear) was measured with an electronic scale with readings accurate to 0.1 kg. The corrected height was derived for every IS patient with Bjure’s formula (log *y* = 0.011*x* − 0.177), where *y* is the loss of trunk height (cm) due to the deformed spine, and *x* is the greatest Cobb angle of the primary curve [31]. Body mass index (BMI) was then calculated, using the standard formula (kilograms per meter squared). Anthropometrical status was defined using BMI for age and sex, using the World Health Organization (WHO) percentile charts [32]. The subjects were defined as underweight (a BMI percentile below the 3rd percentile), normal weight or overweight (a BMI percentile above the 85th percentile). To adjust the anthropometrical status for sex and age, BMI *z*-score, expressed as a number of standard deviations (SD) from the value of the 50th percentile, was calculated. BMI *z*-scores were derived using WHO AnthroPlus, version 1.0.4 (based on World Health Organization growth references) [32]. Waist and hip circumferences were measured midway between the lower rib margin and the iliac crest in the standing position and Waist/Hip Ratio (WHR) and Waist/Height Ratio (WHtR) were calculated.

### 2.4. Body Composition Analysis

Body composition parameters: fat mass (FAT), fat-free mass (FFM), and predicted muscle mass (PMM) were assessed (in kilograms (kg) or as percentage of body weight (%)) based on bioelectrical impedance using a segmental body composition analyzer (BC-418MA Tanita Europe BV, Hoofddorp, The Netherlands). Patients were asked to avoid caffeine and physical activity 12 h prior to the test, avoid all food 4 h prior to the test, and drink two glasses of water 3 h prior to the test. In girls, the procedure was taken within the 10th–15th day of the menstrual cycle.

### 2.5. Ethical Considerations

The study was approved by the Ethics Committee of the Medical University of Silesia. All participants and/or their caregivers gave informed consent. Patient rights were also approved according to the Helsinki Declaration.

### 2.6. Statistical Analysis

The normal distribution of all the variables was confirmed by the Kolmogorow–Smirnov test. Comparisons of categorical variables were performed with a chi-square test. Differences in continuous variables between different scoliosis severity subgroups were assessed by unpaired Student’s *t*-test for independent variables with non-equal variances. One-way analysis of variance (ANOVA) was used to analyze any significant difference among the three anthropometrical subgroups, *i.e.*, underweight, normal weight and overweight. Multivariate regression analysis was performed to identify the variables that influence the curve severity in every anthropometrical subgroup. All statistical analysis was made with the Statistica™ 10 PL software and *p*-value less than 0.05 was considered statistically significant. All results were reported as means ± standard deviations (SD).

## 3. Results

The mean Cobb’s angles in the moderate (*n* = 221) and severe (*n* = 58) groups were 19.96° ± 7.92° and 52.36° ± 12.54°, respectively. Table 1 compares demographic data and anthropometric variables among the moderate and severe subgroups. The incidence of underweight and overweight did not differ significantly between the moderate and severe subgroups. The corrected body height, body weight and BMI were significantly higher in the severe IS subgroup than in the moderate subgroup. However, the corrected body height *z*-score and BMI *z*-score, which are age and sex adjusted parameters, were not significantly different. Interestingly, significant differences in body composition were observed, with higher FAT and lower FFM and PMM in the severe IS subgroup.

For the next stage of the analysis, the study group was further divided according to the anthropometrical status *i.e.*, underweight (*n* = 47/16.8%), normal weight (*n* = 210/75.3%) and overweight (*n* = 22/7.9%).

**Table 1:** Characteristics and comparison of the anthropometric measurements among the moderate and severe groups in total studied idiopathic scoliosis (IS) population.

	Moderate Group (Cobb’s Angle 10°–39°) *n* = 221	Severe Group (Cobb’s Angle ≥40°) *n* = 58	*p* Value
**Age (years)**	14.08 ± 2.9	14.73 ± 2.0	NS
**Sex (M/F)**	46/176	9/49	NS
**Anthropometrical status (U/N/O)**	41(18.6%)/161(72.8%)/19(8.6%)	6(10.3%)/49(84.5%)/3(5.2%)	NS
**Corrected height (cm)**	162.21 ± 13.54	166.54 ± 6.96	<0.05
**Corrected height *Z* score (SD)**	0.84 ± 0.99	0.89 ± 0.89	NS
**Weight (kg)**	48.95 ± 12.54	53.26 ± 9.17	<0.05
**BMI (kg/m^2^)**	18.3 ± 2.85	19.19 ± 3.1	<0.05
**BMI *Z* score (SD)**	−0.52 ± 1.48	−0.4 ± 1.16	NS
**WHR**	0.83 ± 0.06	0.82 ± 0.06	NS
**W/HtR**	0.44 ± 0.05	0.45 ± 0.05	NS
**FAT (%)**	21.21 ± 6.12	23.16 ± 6.98	<0.05
**FFM (%)**	78.78 ± 6.14	76.83 ± 6.96	<0.05
**PMM (%)**	74.95 ± 6.05	73.11± 6.68	<0.05
**TBW (%)**	57.64 ± 4.48	56.19 ± 5.12	<0.05

Data are expressed as mean ± standard deviation and compared using student’s *t*-test and Chi square test.

The comparison of anthropometric parameters in moderate and severe IS subjects in each anthropometrical subgroup are presented in Table 2. There were no significant differences in the underweight subgroup. The corrected height and weight were significantly higher in severe IS subjects in the normal weight subgroup. Overweight and severe IS patients had significantly higher adiposity level assessed by both standard anthropometry (BMI, BMI *z*-score and W/HtR) and body composition analysis (FAT, PMM). In normal weight subjects body composition parameters (FAT, FFM and PMM) differed significantly in the same manner as in the overweight subgroup.

**Table 2:** Comparison of anthropometric parameters among moderate and severe groups depending on anthropometrical status.

	*UNDERWEIGHT*		*NORMAL WEIGHT*		*OVERWEIGHT*	
	*Moderate IS n = 41*	*Severe IS n = 6*	*Moderate IS n = 161*	*Severe IS n = 49*	*Moderate IS n = 19*	*Severe IS n = 3*
***Corrected height (cm)***	164.49 ± 14.75	165.95 ± 5.77	161.6 ± 13.19	167.04 ± 6.8 **	162.48 ± 13.93	159.57 ± 12.23
***Corrected height Z score (SD)***	0.84 ± 0.93	0.3 ± 0.54	0.75 ± 0.98	0.95 ± 0.92	1.6 ± 0.91	1.32 ± 0.82
***Weight (kg)***	42.52 ± 9.62	42.25 ± 4.13	48.87 ± 11.2	53.18 ± 6.36 *	63.45 ± 16.84	76.53 ± 14.2
***BMI (kg/m^2^)***	15.42 ± 1.34	15.33 ± 1.02	18.42 ± 2.15	19.01 ± 1.49	23.54 ± 2.59	29.85 ± 1.15 ***
***BMI Z score (SD)***	−2.14 ± 0.44	−2.5 ± 0.63	−0.37 ± 1.29	−0.34 ± 0.61	1.67 ± 0.29	2.78 ± 0.13 ***
***WHR***	0.81 ± 0.04	0.78 ± 0.04	0.82 ± 0.06	0.82 ± 0.06	0.89 ± 0.05	0.86 ± 0.01
***WHtR***	0.44 ± 0.05	0.44 ± 0.05	0.44 ± 0.04	0.45 ± 0.04	0.53 ± 0.04	0.58 ± 0.02 *
***FAT (%)***	15.88 ± 5.13	13.55 ± 5.42	21.47 ± 4.84	23.4 ± 5.12 *	30.59 ± 5.75	38.47 ± 7.37 *
***FFM (%)***	84.13 ± 5.12	86.41 ± 5.4	78.5 ± 4.86	76.6 ± 5.11 *	69.67 ± 6.33	61.52 ± 7.36
***PMM (%)***	80.24 ± 5.92	82.24 ± 5.01	74.63 ± 4.66	72.89 ± 4.93 *	66.19 ± 5.56	58.37 ± 6.95 *

Data are expressed as mean ± standard deviation and compared using student’s *t*-test: * *p* < 0.05; ** *p* < 0.01; *** *p* < 0.001.

**Figure 1:**
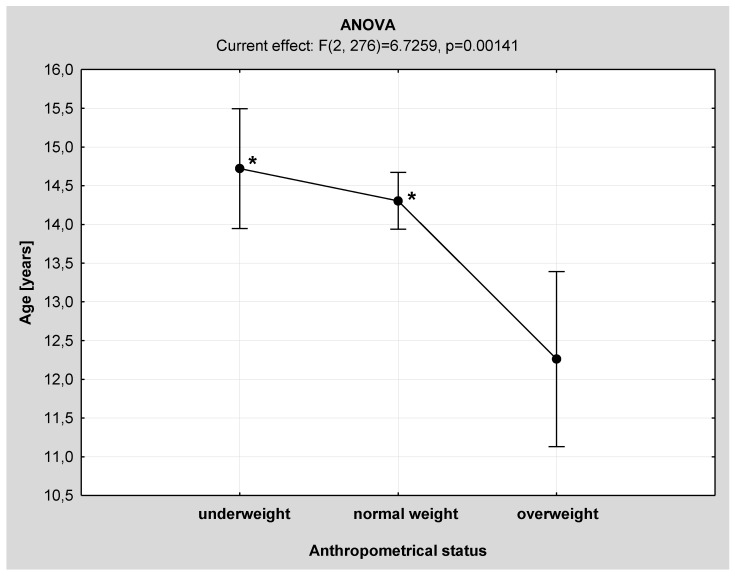
Comparison of chronological age among the anthropometrical subgroups by one-way analysis of variance (ANOVA). * *p* < 0.01 *vs.* overweight subgroup.

**Figure 2:**
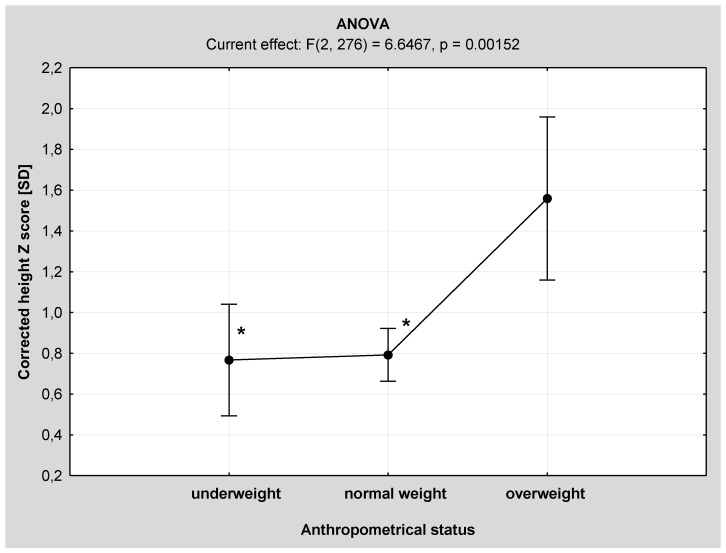
Comparison of corrected height *z*-score among the anthropometrical subgroups by one-way analysis of variance (ANOVA). * *p* < 0.01 *vs.* overweight subgroup.

One-way analysis of variance (ANOVA) revealed significant differences in age and corrected height *z*-scores between the three anthropometrical subgroups (Figure 1 and Figure 2). Overweight IS patients were significantly younger and taller than those from the underweight and normal weight subgroups.

Curve severity, expressed as Cobb’s angle, was the lowest in the normal weight subgroup, although the difference was not statistically significant (23.36° ± 10.55° *vs.* 27.92° ± 16.9° *vs*. 21.95° ± 15.24° in underweight, normal weight and overweight subgroup, respectively).

Multiple regression analysis using a stepwise model was performed on the curve severity with respect to the anthropometrical data. Body composition parameters (FAT, FFM and PMM) were found to be significantly and independently associated with curve severity in the entire studied IS population (Table 3).

**Table 3:** Multivariate regression analysis of the variables influencing the curve magnitude of total studied population.

Variable	Cobb’s Angle		
	Coefficient	Significance	Adjusted *R*^2^
**FAT (%)**	0.204	*p* < 0.001	0.067
**FFM (%)**	−0.205	*p* < 0.001	0.067
**PMM (%)**	−0.188	*p* < 0.01	0.061

The same significant relations were observed in the normal weight and overweight subgroups. Moreover, Cobb’s angle was positively correlated with WHtR and BMI *z*-score in the normal weight and overweight subgroups. In contrast, BMI *z*-score was significantly but negatively associated with Cobb’s angle severity in underweight IS patients (Table 4).

**Table 4:** Multivariate regression analysis of the variables influencing the curve magnitude among different anthropometrical status.

Variable	Coefficient	Cobb’s Angle	Adjusted *R*^2^
**UNDERWEIGHT (*n* = 47)**			
**BMI *Z*-score(SD)**	−0.346	*p* < 0.05	0.138
**NORMAL WEIGHT (*n* = 210)**			
**WHtR**	0.226	*p* < 0.01	0.125
**FAT (%)**	0.200	*p* < 0.01	0.125
**FFM (%)**	−0.196	*p* < 0.01	0.124
**PMM (%)**	−0.186	*p* < 0.01	0.120
**OVERWEIGHT (*n* = 22)**			
**BMI *Z*-score(SD)**	0.598	*p* < 0.01	0.290
**FAT (%)**	0.484	*p* < 0.05	0.154
**FFM (%)**	−0.473	*p* < 0.05	0.142
**PMM (%)**	−0.500	*p* < 0.05	0.172

## 4. Discussion

This large scale cross-sectional study confirmed the significant relationship between spinal deformity and anthropometrical status in IS children as previously published in our preliminary report [8]. Body composition was significantly more disturbed in severe than in moderately IS affected patients. Further multivariate regression analysis confirmed that Cobb’s angle was still independently associated with variations of the body composition parameters (FAT, FFM and PMM) and age and sex adjusted standard anthropometrical parameters (W/HtR and BMI *z*-score). Interestingly, spinal deformity correlates with FAT, FFM, PPM, in the same positive manner in both overweight and normal weight subgroups, whilst in underweight IS children, a significant correlation was found only for the BMI *z*-score and W/Ht but in the opposite negative way.

Body composition analysis by BIA has only been used in two studies: our preliminary report [8] and a study by Ramirez *et al.* [33] in a group of 27 girls with adolescent IS (AIS) surgery candidates, with a large age distribution (13–26 years). Ramirez *et al.* observed that body composition parameters were significantly lower in the AIS patients than in healthy controls, although this may be attributable to the very high degree of underweight (55.6% with BMI less than 18.5) and the severity of spinal deformity (mean max. Cobb’s angle was 66°) [33]. 

The results of a study by Barrios *et al.* analyzing body composition in girls with IS based on skin folds measurement and somatotype assessment (endomorphic—adipose tissue, mesmorphic—muscular tissue, and ectomorphic—bone tissue) showed that girls with IS had less adipose tissue than controls, although the difference was not statistically significant (*p* < 0.06) [22]. The impact of body components on the grade of scoliosis severity was not evaluated in either of the two studies.

The recently published first prospective cohort study showed that altered body composition (low FAT, FFM and BMI) before the onset of clinically detected spinal deformity is a risk factor for IS [9]. Our data confirmed these findings only in respect of low FFM in all the studied children. However, the positive correlation between adiposity level and spinal deformity in normal weight and overweight children, which confirmed our previous results [8], may be associated with the excessive gain of adipose tissue during the progression of spinal deformity. Milder forms of IS in overweight children may be underestimated due to anatomical/technical reasons. Further prospective studies are warranted to confirm these hypotheses.

Although underweight was two-fold more prevalent than overweight in our group of patients, spinal deformity subgroups did not differ with regard to weight prevalence. The majority of previously published studies focusing on the anthropometrical status of IS children showed that the BMI of IS children is significantly lower than in the general population [11,12,13,22,23,25]. The use of BMI as the only marker of anthropometrical status in children may cause an important bias. A recent analysis of 3631 children revealed a significantly higher percentage of underweight boys than girls; however, the cut-off points for BMI ranges used in the analysis were applicable to adults, thus resulting in a significant underestimation of overweight and obesity and overestimation of underweight in the studied population [24]. BMI adjustment for age and sex seems to be especially important as some papers state that the disproportion in the anthropometrical status between IS children and the general population rises with age [22,23]. In our study, we used BMI *z*-score that was similar in both subgroups categorized for the spinal deformity degree. The BMI *z*-score was significantly associated with scoliosis severity in either the underweight and overweight subgroups but not in the normal weight subjects.

Abnormal growth pattern is considered an etiological model of IS development, especially during puberty. Nicolopoulos *et al.* in their study showed that girls with AIS had significantly different silhouette and its components than controls: taller height, sedentary height and longer lower extremities [5]. Similarly, in a study by Cheung *et al.* encompassing 598 girls with IS, girls in the prepubertal period (Tanner I) were significantly shorter and had shorter sedentary height and between-arms distance compared to the control group; with the progression of puberty (from Tanner II to IV), the height, sedentary height and between-arms distance were significantly greater in girls with IS [23]. However, these studies did not compare growth pattern markers within the IS group in respect of the severity of spinal deformity. In our study, corrected body height was significantly higher than in the moderate IS subgroup. However, after the adjustment for sex and age by the *z*-score calculation, this parameter was no longer significantly different. Further analysis within the anthropometrically different subgroups revealed that corrected height was significantly higher only in normal weight children with severe IS. Abnormal pattern of growth is a common finding in children with IS. Leptin, produced mainly by the adipose tissue, is a hormonal factor, which influences both growth and bone mineralization. In the study by Qiu *et al.*, the leptin level was significantly lower in girls with AIS than in controls and correlated with bone mineral density [3]. Although these were not measured in the current study, other authors emphasize that the role of leptin in the pathogenesis of scoliosis may be associated with the important difference between spine growth velocity compared to the extremities [1,4,6,34] and a lower bioavailability [35]. These hypotheses may partially explain the pathogenesis of IS in underweight patients. In our study based on the ANOVA analysis, overweight IS children were significantly younger and taller than children with another anthropometrical status. Moreover, hyperleptinemia is a well known phenomenon in the obese children [36]. An earlier onset of puberty and peak height velocity in obese children seems a plausible pathophysiological explanation [37]. Factors which have been implicated in the accelerated growth in obese children include increased leptin and insulin levels, adrenal androgens and insulin-like growth factor-1 (IGF-1) [38]. Hence, it is possible that more than one anthropometrical phenotype and etiological model of idiopathic scoliosis exist. As obesity in children and young people is a serious and growing problem in both the USA and Europe [15,16,17,18,19], the stereotypical image of a scoliotic child as a thin person needs to be partially challenged.

Overweight and obesity in people with IS tend to be considered in the aspect of postoperative long-term results of a surgical procedure on the scoliotic curve. Chen *et al.* reported larger thoracic and thoraco-lumbar kyphosis curve in overweight or obese IS adults and a higher prevalence of hypertension [39]. The patients also had worse Visual Analogue Scale (VAS) test results assessing disability and pain tolerance level, both pre- and post-operatively. However, the results of surgical treatment of scoliosis (measured by curve severity) in this group of patients were no worse than normal weight patients [39]. Upasani *et al.* conducted a similar study in a group of 241 adolescents (48 overweight) with IS and found no relationship between the nutritional status (BMI) and the results of surgical treatment of scoliosis [40]. Another study, based on group of 427 patients with IS showed, that girls with larger curve severity (Cobb’s angle >50°) were older and had higher BMI than patients with milder deformity [21]. The recent study by Hardesty *et al.* confirmed that obesity negatively affects spinal surgery in IS. Increased BMI correlated with increased operative time, intraoperative blood loss, amount of intraoperative crystalloids, and difficulty with administration of spinal anesthesia [41].

The waist/height ratio (WHtR) seems to be a useful parameter for anthropometrical evaluation of the severity of deformity in children with IS. In our study, WHtR correlated significantly with spinal deformity in the normal weight and overweight subgroups. WHtR is now widely studied with the aim to find relatively simple parameters of fat tissue distribution in connection with visceral obesity and its comorbidities. A recent analysis showed that WHtR is better than the waist/hip ratio (WHR) for the prognosis of visceral obesity and its comorbidities [42,43,44]. However, waist circumference measurement may be technically difficult, especially in underweight children with very severe scoliosis in the lumbar spine. Therefore, the usefulness of WHtR as a prognostic parameter in idiopathic scoliosis needs to be further investigated.

The major limitations of our study are the cross-sectional design and a relatively low number of overweight patients. Nevertheless, the obtained differences after multifactorial adjustments were still statistically significant. Furthermore, to our knowledge, there are no other publications concerning the relationship between spinal deformity and body composition based on BIA in IS patients. Further research in that area of idiopathic scoliosis is warranted, especially in the subgroup of overweight children with IS.

## 5. Conclusions

The degree of both being underweight and overweight in IS patients is significantly related to the scoliotic curve severity but in the opposite manner. Even in normal weight IS patients, body composition is associated with the severity of the disease. Overweight IS children are significantly younger and taller than other anthropometrical IS subgroups. Body composition analysis by BIA seems to be a useful tool to provide baseline characteristics of both normal weight and overweight IS children and may be used as the follow-up procedure if the dietary intervention is needed. In the standard anthropometrical analysis, the most valuable parameters are waist to height ratio (WHtR) in normal weight IS subjects and BMI *z*-score in underweight and overweight patients. Based on the current study, the Cobb’s angle variability is related to body composition in 6.1%–17.1% only. This is why further prospective studies concerning body composition (based also on other techniques), especially in relation to deformity progression in children and adolescents with IS, are warranted.

## Abbreviations

The following abbreviations are used in this manuscript:

IS: Idiopathic Scoliosis
BMI: Body Mass Index
BIA: Bioelectrical Impedance Analysis
WHR: Waist Hip Ratio
WHtR: Waist Height Ratio
FAT: Fat Mass
FFM: Fat Free Mass
PMM: Predicted Muscle Mass
TBW: Total Body Water
U/N/O: underweight/normal weight/overweight
